# Effects of systemic glucocorticosteroids on peripheral neutrophil functions in asthmatic subjects: an *ex vivo* study

**DOI:** 10.1155/S0962935195000408

**Published:** 1995-07

**Authors:** P. L. Paggiaro, L. Bancalari, D. Giannessi, W. Bernini, G. Lazzerini, R. Sicari, E. Bacci, F. L. Dente, B. Vagaggini, R. De Caterina

**Affiliations:** 1 2nd Institute of Internal Medicine Respiratory Pathophysiology, and CNR Institute of Clinical Physiology Pisa Italy

## Abstract

In 21 asthmatic subjects, several functions of isolated peripheral neutrophils (chemokinesis and chemotaxis toward 10% *E. coli*; superoxide anion generation after PMA; leukotriene B_4_ (LTB_4_) release from whole blood and isolated neutrophtls, before and after different stimuli) were evaluated during an acute exacerbation of asthma, and after 14 – 54 days of treatment with systemic glucocorticosteroids (GCS). During acute exacerbation, superoxide anion generation was higher in asthmatics than in eleven normal subjects (39.2 ± 14.1 *vs.* 25.2 ± 7.3 nmol, p < 0.05); there was a significant correlation between FEV_1_ (% of predicted) and neutrophil chemotaxis (r = −0.52, p = 0.04). After treatment, there was no significant change in all neutrophil functions, except for a decrease in neutrophil chemotaxis in subjects who showed an FEV_1_ increase > 20% after GCS treatment (from 131 ± 18 to 117 ± 21 μm, p = 0.005). Chemokinesis sicantly decreased in all subjects, and the changes significantly correlated with an arbitrary score of the total administered dose of GCS (r = 0.57, p < 0.05). These data suggest that neutrophil activation plays a minor role in asthma, and that treatment with GCS is not able to modify most functions of peripheral neutrophils in asthmatic subjects; chemotaxis seems to be related only to the severity of the asthma and it could reflect the improvement of the disease.


Mediators of Inflammation 4, 251–256 (1995)

### Effects of systemic glucocorticosteroids on peripheral neutrophil functions in asthmatic subjects: an *ex vivo* study

P. L. Paggiaro,[CA] L. Bancalari, D. Giannessi, W. Bernini, G. Lazzerini, R. Sicari, E. Bacci, F. L. Dente, B. Vagaggini and R. De Caterina

2nd Institute of Internal Medicine, Respiratory Pathophysiology, and CNR Institute of Clinical Physiology, Pisa, Italy

[CA]Corresponding Author

**In 21 asthmatic subjects, several functions of isolated peripheral neutrophils (chemokinesis and chemotaxis toward 10% *E. coli*; superoxide anion generation after PMA; leukotriene $B_4$ ($LTB_4$) release from whole blood and isolated neutrophils, before and after different stimuli) were evaluated during an acute exacerbation of asthma, and after 14 – 54 days of treatment with systemic glucocorticosteroids (GCS). During acute exacerbation, superoxide anion generation was higher in asthmatics than in eleven normal subjects (39.2 ± 14.1 *vs.* 25.2 ± 7.3 nmol, $p < 0.05$); there was a significant correlation between $FEV_1$ (% of predicted) and neutrophil chemotaxis ($r = -0.52$, $p = 0.04$). After treatment, there was no significant change in all neutrophil functions, except for a decrease in neutrophil chemotaxis in subjects who showed an $FEV_1$ increase >20% after GCS treatment (from 131 ± 18 to 117 ± 21 μm, $p = 0.005$). Chemokinesis significantly decreased in all subjects, and the changes significantly correlated with an arbitrary score of the total administered dose of GCS ($r = 0.57, p < 0.05$). These data suggest that neutrophil activation plays a minor role in asthma, and that treatment with GCS is not able to modify most functions of peripheral neutrophils in asthmatic subjects; chemotaxis seems to be related only to the severity of the asthma and it could reflect the improvement of the disease.**

**Key words:** Asthma, Chemotaxis, Glucocorticosteroids, $LTB_4$, Neutrophils

### Introduction

Asthma has been defined as reversible airway obstruction, associated with nonspecific bronchial hyperresponsiveness, which is sustained by a chronic airway inflammation.[1] Pathology of fatal asthma[2] and direct *in vivo* measurements of indices of airway inflammation by bronchoalveolar lavage (BAL) and bronchial biopsy[3,4] has confirmed the major role played by the recruitment of inflammatory cells in the airways of subjects with asthma of different severity.

While eosinophils are pivotal cells in asthma, the role of neutrophils in the pathophysiology of asthma is still uncertain. Neutrophil counts are increased in bronchial (BL) and bronchoalveolar lavage (BAL) of animals and humans after acute exposure to allergens, chemicals (like toluene diisocyanate, TDI) or oxidants (like ozone);[5–7] but in the stable phase, asthmatics show neutrophil counts in BAL or in bronchial biopsy that are not different from those found in normal subjects.[8,9] Markers of activation of neutrophils have been inconsistently demonstrated in the blood of asthmatic subjects. Peripheral neutrophils of asthmatic subjects seem to have higher releasability of mediators,[10–15] higher superoxide anion generation[16] and higher migratory activity[11] than in normal subjects. However, the differences between asthmatic and normal subjects are mild; the observed abnormalities are not strictly related to the severity of asthma and they do not change significantly with the treatment of asthma. Thus, the significance of these abnormalities in the pathophysiology of the disease has not been demonstrated. Since neutrophils contain many destructive proteases and are able to release arachidonic acid metabolites, such as the potent chemoattractant leukotriene $B_4$ ($LTB_4$), these cells can be considered to play a pathologic role in asthma.

Glucocorticosteroids (GCS) and other anti-inflammatory drugs now represent the first choice in the treatment of asthma.[1] The efficacy of inhaled or systemic GCS can be evaluated by the changes in pulmonary function tests and bronchial reactivity, but also by the improvement in the markers of airway inflammation.[17] GCS can

© 1995 Rapid Science Publishers modify several functions of the inflammatory cells involved in asthma, and these effects can explain the efficacy of these drugs in asthma treatment. Some *in vitro* studies have shown that GCS can stabilize lysosomes, inhibit chemotaxis and other neutrophil functions,[17–21] but these effects occur at very high concentrations of GCS, and their clinical significance has not been proven. Few *in vivo* or *ex vivo* studies have evaluated the effect of physiologically important concentrations of GCS on neutrophil functions, showing effects on some[19–21] but not on other functions.[18,22]

The aim of our study was to assess whether treatment with systemic GCS in subjects with exacerbation of asthma was able to modify several functions of peripheral neutrophils potentially involved in the pathophysiology of asthma, such as chemotaxis, release of oxygen radicals and $LTB_4$. Furthermore, the effect of GCS treatment on these neutrophil functions was related to the improvement in the airway obstruction as assessed by spirometry.

### Subjects

Twenty-one non- or ex-smoker subjects (seven male and 14 female, with a mean age of $42 \pm 16$ years) with moderate to severe bronchial asthma were studied. Asthma diagnosis has been made in the past on the basis of: (1) clinical history of wheeze, cough or shortness of breath; (2) increase in $FEV_1 > 12\%$ after acute administration of inhaled $beta_2$-agonists or after a short term course of intensive treatment with bronchodilators and GCS;[23] or (3) methacholine $PD_{20}FEV_1$ lower than 1 mg. The mean duration of asthma was $11 \pm 9$ years. At the time of the first examination, all subjects were under regular treatment with bronchodilators (inhaled $beta_2$-agonists and/or oral theophylline) and inhaled anti-inflammatory drugs (sodium cromoglycate or nedocromil sodium in eight subjects, beclomethasone dipropionate 800 – 1500 μg daily in 13 subjects). The aetiology of asthma was allergic in eight subjects, occupational in two subjects, and intrinsic in the remaining eleven subjects.

The first examination was done when subjects experienced a spontaneous exacerbation of asthma, with increase in asthma symptoms despite their current treatment. At this time, they performed spirometry and collected a blood sample to measure total and differential white blood cells (WBC), migratory activity and other markers of activity of peripheral neutrophils. Systemic GCS were added to their regular treatment: 6-methyl-prednisolone (Urbason, Hoechst, Italy) 40 mg/day for 3 days and then 20 mg/day by i.m or oral route, or equivalent doses of deflazacort (Flantadin, Lepetit, Italy). They were examined every 2 weeks until a significant improvement in asthma symptoms occurred. At the end of the treatment period, they repeated pulmonary function tests and blood measurements.

In the same time, eleven normal subjects from our Unit team were examined as regards the spontaneous and 20 μM calcium ionophore A23187-induced $LTB_4$ release from whole blood, migratory activity and superoxide anion generation of isolated peripheral neutrophils.

### Methods

*Pulmonary function tests:* Expiratory flow-volume curves were performed by means of a HP Pulmonary Desk System 47804/A; three acceptable manoeuvres were obtained each time, according to ATS criteria.[23]

*Haematology:* Total and differential WBC count in the blood was measured with a laser Coulter (H1 Bayer, Basel, Switzerland).

For the release of $LTB_4$ from whole blood, heparinized whole blood was incubated for 30 min at 37°C in the presence of either 20 μM calcium ionophore A 23187 (Sigma, St. Louis, MO, USA), or 0.2 mM *N*-formyl-methionyl-leucyl-phenylalanine (f-MLP, Sigma) or 300 μg/ml zymosan (ZAS, Sigma). Control incubations were performed in the absence of stimuli. After incubation, serum was obtained by centrifugation at $1500 \times g$ for 10 min and divided into aliquots for subsequent eicosanoid assay. Leukotriene $B_4$ ($LTB_4$) concentration in the supernatant was measured using radioimmunoassay (RIA) with a tritiated tracer (Amersham, UK); an $LTB_4$ standard was purchased from Upjohn (Kalamazoo, MI, USA); and the specific antiserum was a gift from Dr Frank Carey and Dr Robert Forder, ICI Pharmaceuticals, UK. The sensitivity of $LTB_4$ RIA was $4.3 \pm 0.9$ pg (coefficient of intra-assay variation: 12%).

*Preparation of isolated neutrophils:* For preparation of granulocytes, ACD-anticoagulated blood (60 ml) was centrifuged at $180 \times g$ for 10 min; the supernatant platelet-rich plasma was discarded, and the remaining aliquot was mixed with a 3.5% solution of dextrane T500 (Pharmacia, Uppsala, Sweden) in saline solution, to a final concentration of 1.5%. After allowing differential cell layering for 60 min at room temperature, leukocyte-rich plasma was collected and centrifuged at $270 \times g$ for 10 min; after discharging the supernatant, the pellet was resuspended in 7.5 ml phosphate-buffered saline without calcium and magnesium (PBS, Sigma) containing 0.5% bovine serum albumin (BSA), carefully layered onto 3.0 ml of Ficoll–Hypaque (Lymphoprep, Nycomed AS, Oslo, Norway) and centrifuged at 350 × **g** for 30 min. Mononuclear cells at the interface between PBS and Ficoll–Hypaque were removed (see below); lysis of red blood cells was performed by adding 6 ml of distilled water to the pellet for 15 s, and osmolarity was re-equilibrated to normal levels with 2 ml of 3.5% NaCl solution. After further centrifugation (270 × **g** for 5 min), the final pellet was resuspended in 1 ml of PBS and cell count performed in a Thoma–Zeiss microscope chamber. Cell viability was evaluated by Trypan blue exclusion and was always >95%. Neutrophil purification, evaluated on a standard May–Grunwald–Giemsa smear, was also >95%.

Purified neutrophils were used to perform measurements of migratory activity, generation of superoxide anion, and release of $LTB_4$ after different stimuli.

*Migratory activity.* Chemokinesis and chemotaxis of granulocytes were measured by a modified Boyden chamber technique. A 0.7 ml aliquot of a cell suspension adjusted to $1 \times 10^6$/ml was put into the upper compartment of a Boyden chamber, using a 13 mm diameter and 3 μm pore size filter (Millipore Co, Bedford, USA). Dulbecco solution or 10% *E. coli* culture supernatant was placed in the lower compartment of the chamber to study chemokinesis (random migration) or chemotaxis, respectively. Incubation was performed for 2 h at 37°C in a $CO_2$ incubator. Filters were fixed on a microscope slide and stained with haematoxylin–eosin. Migration of granulocytes was measured according to the 'leading-front' technique on ten randomly selected low power (×40) fields. All samples were processed in triplicate. The intra-assay and inter-assay variations were 7% and 13%, respectively.

*Generation of superoxide anion.* Generation of superoxide anion was tested by incubating 0.7 ml of a suspension of granulocytes at $2.1 \times 10^6$ cells/ml for 15 min at 37°C in a shaking waterbath with 50 μl of 30 mg/ml cytochrome C (Sigma) and 0.75 ml of 2 μg/ml prewarmed phorbol myristate acetate (PMA, Sigma). In order to subtract any $O_2$-dependent change in adsorbance, the same assay was also carried out in the presence of 10 μl of 3 mg/ml superoxide dismutase. The reaction was stopped by placing the tubes in ice, and the suspension was centrifuged at 1500 × **g** for 5 min at 4°C to remove cells. The reduction of cytochrome C was measured by reading the adsorbance of the supernant at 550 nm wavelength. The intra-assay variation was 4.9%.

*Release of $LTB_4$ after different stimuli.* A suspension of isolated granulocytes (concentration, $7.5 \times 10^6$ cells/ml) was incubated for 30 min at 37°C in the presence of either 20 μM calcium ionophore A 23187, or 0.2 mM *N*-formyl-methionyl-leucyl-phenylalanine, or 300 μg/ml zymosan. Control incubations were performed in the absence of stimuli. At the end of the incubation, a supernatant was obtained by centrifugation at 1500 × **g** for 10 min and divided into aliquots for subsequent eicosanoid assay. The $LTB_4$ concentration in the supernatant was measured using the same radioimmunoassay (RIA) as described previously.

*Statistical analysis:* Data are reported as mean ± standard deviation (S.D.). Paired and unpaired Student's *t*-tests were performed when appropriate. Regression analysis was used to correlate pulmonary function tests with indices of neutrophil activity in the blood. A level of significance lower than 5% was considered significant.[24]

### Results

At the time of asthma exacerbation, all subjects were currently symptomatic with a moderate to severe airway obstruction (mean $FEV_1$, 1.55 ± 0.48 l, 51.1 ± 15.1% of the predicted value). Haematology showed high eosinophil count (>400 cells/μl) in nine of 21 subjects (43%). The evaluation of the peripheral neutrophil functions in the asthmatic subjects showed no significant difference with respect to normal subjects as regards the spontaneous and 20 μM calcium ionophore A23187-induced $LTB_4$ production from whole blood (2.1 ± 4.7 μg/ml and 152.2 ± 72.8 μg/ml in asthmatics, and 2.0 ± 3.4 μg/ml and 93.1 ± 23.8 μg/ml in normal subjects, respectively) and chemotaxis of isolated peripheral neutrophils (122.0 ± 16.4 μm in asthmatics, and 117.5 ± 10.1 μm in normal subjects). Generation of superoxide anion from isolated peripheral neutrophils was significantly higher in asthmatics (39.2 ± 14.1 nmol) than in normal subjects (25.2 ± 7.3 nmol, $p < 0.05$), and chemokinesis showed a trend in increasing in asthmatic subjects in comparison with normal subjects (91.6 ± 15.6 μm *vs.* 86.4 ± 6.8 μm, $p = 0.07$).

In asthmatic subjects there was a mild significant inverse relationship between $FEV_1$ (in %

of the predicted value) and neutrophil chemotaxis ($r = -0.52, p = 0.04$).

At the end of the treatment with systemic GCS, when symptoms of exacerbation were recovered in all subjects, mean $FEV_1$ and FVC significantly increased with respect to the pre-treatment values (Table 1). Haematology showed a significant increase in total WBC and neutrophil count, and a significant decrease in eosinophil count. A significant reduction of neutrophil chemokinesis was observed after treatment. No change was observed in chemotaxis and superoxide anion generation from isolated peripheral neutrophils (Table 1).

The spontaneous and induced release of $LTB_4$ from whole blood and from isolated peripheral neutrophils was not significantly reduced after treatment with GCS (Table 2). There was a significant increase in $LTB_4$ release from whole blood after 20 μM calcium ionophore incubation (from 152.2 ± 72.8 to 204.7 ± 89.3 μg/ml, $p < 0.001$) and a trend for ZAS-induced $LTB_4$ release (from 48.1 ± 20.8 to 60.0 ± 27.4 μg/ml, $p = 0.08$); this increase was not observed when

Table 1. Mean values (± SD) of pulmonary function tests, blood cell count, migratory activity and superoxide anion generation of peripheral isolated neutrophils in 21 asthmatic subjects, before and after treatment with systemic GCS

| Parameter | Before treatment ($n$ = 21) | After treatment ($n$ = 21) |
|---|---|---|
| FVC (%pred) | 74.4 ± 16.3* | 86.9 ± 19.5 |
| $FEV_1$ (%pred) | 51.1 ± 15.1* | 68.0 ± 23.6 |
| White blood cells (cells/μl) | 7 470 ± 2 757* | 8 926 ± 2 636 |
| Neutrophils (cells/μl) | 4 364 ± 2 662* | 5 660 ± 2 862 |
| Eosinophils (cells/μl) | 501 ± 428* | 224 ± 209 |
| Neutrophil chemokinesis (μm) | 91.6 ± 15.6* | 82.2 ± 12.5 |
| Neutrophil chemotaxis (μm) | 122.0 ± 16.4 | 118.6 ± 14.0 |
| Superoxide anion generation (nmol) | 45.4 ± 23.2 | 43.8 ± 22.5 |

*$p < 0.05$ between before and after treatment.

Table 2. Mean values (± SD) of $LTB_4$ release from whole blood and from isolated peripheral neutrophils in 21 asthmatic subjects, before and after treatment with systemic GCS

| Stimulus | Before treatment ($n$ = 21) | After treatment ($n$ = 21) |
|---|---|---|
| Whole blood-$LTB_4$ release (μg/ml) | | |
| No stimulus | 2.1 ± 4.7 | 1.3 ± 1.2 |
| 20 μM calcium ionophore A23187 | 152.2 ± 72.8* | 204.7 ± 89.3 |
| FMLP | 3.9 ± 4.1 | 4.2 ± 3.6 |
| ZAS | 48.1 ± 20.8* | 60.0 ± 27.4 |
| Isolated neutrophils-$LTB_4$ release (μg/ml) | | |
| No stimulus | 0.5 ± 0.6 | 0.5 ± 0.9 |
| 20 μM calcium ionophore A23187 | 50.0 ± 42.3 | 56.9 ± 32.6 |
| FMLP | 2.3 ± 2.9 | 2.3 ± 2.2 |
| ZAS | 1.0 ± 2.4 | 0.6 ± 0.7 |

*$p < 0.05$ between before and after treatment values.

$LTB_4$ release was obtained from isolated neutrophils.

At the end of the treatment with systemic GCS, ten subjects showed an increase in $FEV_1$ greater than 20% with respect to the pre-treatment evaluation; they were considered as responders. The comparison between responders and non-responders as regards haematology and indices of neutrophil functions at the time of asthma exacerbation showed that eosinophils were higher in responders than in non-responders (Table 3). No change in neutrophil functions was observed after treatment in both groups, except for a significant decrease in chemotaxis of isolated peripheral neutrophils in responders (from 130.6 ± 17.9 to 117.0 ± 20.6 μm, $p = 0.005$) but not in non-responders. Total WBC and neutrophil counts increased after treatment, but the changes were statistically significant only in non-responders; eosinophils decreased significantly in responders, and showed a trend ($p = 0.09$) to decrease in non-responders. No difference between responders and non-responders could be observed as regards the dose of oral GCS and the duration of treatment (22.8 ± 10.8 days in responders *vs.* 24.1 ± 12.6 days in non-responders).

An arbitrary score of the total dose of GCS administered during all periods of treatment (daily dose of 6-methyl-prednisolone or equivalent dose of deflazacort × number of days of treatment) was computed for each patient. Responders had a similar score to non-responders. This score was not significantly related to changes in the haematology or indices of neutrophil functions; there was only a significant relationship between this score and the decrease after treatment in chemokinesis from isolated peripheral neutrophils ($r = 0.57, p < 0.05$).

There was no difference in the changes of indices of neutrophil activity between subjects who received regular $beta_2$-agonists in addition to systemic GCS treatment and subjects who did not, nor between subjects who were previously treated with inhaled GCS and those treated with cromones.

### Discussion

We showed that treatment with systemic GCS, at doses able to induce a significant improvement in pulmonary function in asthmatic subjects, was able to cause minimal effects on some indices of activity of peripheral blood neutrophils. Chemotaxis towards *E. coli* endotoxin was the only marker of neutrophil activity which significantly reduced after GCS in subjects who

Table 3. Mean values (± SD) of haematology, migratory activity and superoxide anion generation from isolated peripheral neutrophils in asthmatic subjects who responded or not to systemic GCS treatment with an $FEV_1$ increase of 20% or more

| Parameter | Responders ($n = 10$) | | Non-responders ($n = 11$) | |
|---|---|---|---|---|
| | Before treatment | After treatment | Before treatment | After treatment |
| $FEV_1$ (%pred) | 48 ± 13 | 80 ± 22* | 54 ± 17 | 57 ± 20 |
| White blood cells (cells/µl) | 8208 ± 3212 | 9686 ± 2547 | 6731 ± 2127 | 8285 ± 2655* |
| Neutrophils (cells/µl) | 4902 ± 3206 | 5915 ± 2902 | 3827 ± 2010 | 5451 ± 2952* |
| Eosinophils (cells/µl) | 680 ± 488 | 271 ± 253* | 323 ± 279** | 185 ± 168 |
| Chemokinesis (µm) | 88.8 ± 18.2 | 79.5 ± 13.2 | 92.9 ± 15.0 | 83.5 ± 12.7 |
| Chemotaxis (µm) | 130.6 ± 17.9 | 117.0 ± 20.6* | 117.7 ± 14.6 | 119.4 ± 10.7 |
| Superoxide anion generation (nmol) | 45.9 ± 33.5 | 44.9 ± 32.3 | 45.0 ± 12.0 | 43.7 ± 11.9 |

*$p < 0.05$ with respect to the pre-treatment evaluation; **$p < 0.05$ with respect to responders.

responded to GCS treatment with an increase in $FEV_1$, but no change in $LTB_4$ release and superoxide anion generation from neutrophils was observed. These data confirm and extend the results obtained by Gin and coworkers[19] on six asthmatic patients; in particular, the effect was obtained only in subjects clinically sensitive to GCS and not in subjects who did not show any significant change in $FEV_1$ after treatment. Therefore, the decrease in neutrophil chemotaxis could be considered as a consequence of GCS treatment on airway inflammation, and not as a nonspecific effect of steroids directly on peripheral blood cells, because the same effect was not observed in subjects who were non-responders to GCS treatment, though they received similar doses of GCS. On the other hand, chemokinesis was affected by GCS in a dose-dependent manner in all subjects, independently from the degree of clinical response, suggesting that this neutrophil function can be directly affected by GCS and not by an effect on the mediators of the asthmatic inflammation.

These results are in contrast with previous reports, suggesting a lack of effect of GCS on neutrophil locomotion in normal and asthmatic subjects,[18,25] but they agree with our previous results obtained in asthmatic subjects tested with high dose inhaled beclomethasone dipropionate for 1 month.[26]

It is not surprising that some asthmatic patients did not show a significant improvement in $FEV_1$ after GCS treatment. It is well known that some patients are resistant to the steroids,[27] and that the effect of GCS on $FEV_1$ can require a different duration of treatment. Although all our patients showed at the diagnosis the typical functional abnormalities of asthma (reversibility and/or bronchial hyperreactivity), it is possible that some of them had a concomitant chronic obstructive pulmonary disease of variable degree which could contribute to the airway obstruction. Patients who were responders to GCS showed, in effect, higher eosinophil counts in the blood than non-responders. Furthermore, the post-treatment evaluation was performed after improvement in symptoms, which could not correspond to an improvement in pulmonary function. However, the duration of treatment and the total administered dose of GCS were equivalent in responders and non-responders, suggesting that the lack of clinical response was not due to an insufficient treatment in non-responders.

The lack of effect of GCS on $LTB_4$ release from whole blood and isolated neutrophils after several stimuli confirm what has been previously reported after a short-term treatment with GCS.[22] $LTB_4$ release from neutrophils could be considered as a way to recruit new cells in the airways after an initiating stimulus; because neutrophils are usual inhabitants of the airways,[28] this function could be physiologically important. Although some methodological problems need to be considered (the dose and the characteristic of the different physiological and non-physiological stimuli to induce $LTB_4$ release from isolated neutrophils), these results confirm a minor role of neutrophil activation in asthma. The higher release of $LTB_4$ from whole blood after GCS is due to the higher number of peripheral neutrophils, induced by the well-known effect of GCS on leukocyte margination. The different increase in $LTB_4$ release in whole blood and in isolated neutrophils after the various stimuli was due to the different amount of neutrophils obtained in the different preparations, and to the presence of platelets in whole blood which can increase the availability of arachidonic acid as a substrate for $LTB_4$ production from neutrophil lypoxygenase.

We must consider, however, that neutrophils of the airways, obtained by bronchial or broncho-alveolar lavage (BAL), could show a higher degree of activation, and a greater modulation in their functions by anti-inflammatory treatment, with respect to blood neutrophils. Considering that several studies showed a good correlation between activation of eosinophils derived from the blood and from BAL fluid,[29] the difference between blood and BAL neutrophils, although hypothetical, is not very probable.

As an additional finding, this study shows that most functions of peripheral neutrophils from asthmatic subjects during asthma exacerbations are not different from those measured in a small group of normal subjects, except for superoxide anion generation. Furthermore, chemotaxis toward *E. coli* endotoxin was significantly correlated with an index of asthma severity like $FEV_1$. The partial disagreement between previous data[10–16] and our observation regarding the increased capability of peripheral neutrophils to generate arachidonic acid metabolites and superoxide anion, could be explained by the small group of normal subjects in our study, by some methodological differences and by the different severity of the disease.

In conclusion, our study shows that neutrophil activation plays a minor role in asthma, and that treatment with systemic GCS is not able to modify most neutrophil functions in asthmatic subjects. Chemotaxis only seems to be related to the severity of asthma and it could reflect the improvement of the disease after GCS treatment. However, further studies on airway neutrophils are needed.

### References

1. NHLBI. International consensus report on diagnosis and treatment of asthma. *Eur Respir J* 1992; **5:** 601–641.
2. Jeffrey PK. Pathology of asthma. *Brit Med Bull* 1992; **48:** 23–39.
3. Djukanovic R, Roche WR, Wilson JW, *et al.* Mucosal inflammation in asthma. *Am Rev Respir Dis* 1990; **142:** 434–457.
4. Beasley R, Roche WR, Roberts JA, Holgate ST. Cellular events in the bronchi in mild asthma and after bronchoprovocation. *Am Rev Respir Dis* 1989; **139:** 806–817.
5. De Monchy JGR, Kauffman HF, Venge P, *et al.* Bronchoalveolar eosinophilia during allergen-induced late asthmatic reactions. *Am Rev Respir Dis* 1985; **131:** 373–376.
6. Fabbri LM, Boschetto P, Zocca E, *et al.* Bronchoalveolar neutrophilia during late asthmatic reactions induced by toluene diisocyanate. *Am Rev Respir Dis* 1987; **136:** 36–42.
7. Fabbri LM, Aizawa H, Alpert SE, *et al.* Airway hyperresponsiveness and changes in cell counts in bronchoalveolar lavage after exposure to ozone in dogs. *Am Rev Respir Dis* 1984; **129:** 288–291.
8. Kirby JG, Hargreave FE, Gleich GJ, O'Byrne PM. Bronchoalveolar cell profiles of asthmatic and nonasthmatic subjects. *Am Rev Respir Dis* 1987; **136:** 379–383.
9. Lozewicz S, Gomez E, Ferguson H, Davies RJ. Inflammatory cells in the airways in mild asthma. *Br Med J* 1988; **297:** 1515–1516.
10. Wang SR, Yang CM, Wang SS, Han SH, Chiang BN. Enhancement of A23187-induced production of the slow-reacting substance on peripheral leukocytes from subjects with asthma. *J Allergy Clin Immunol* 1986; **77:** 465–471.
11. Radeau T, Chavis C, Damon M, *et al.* Enhanced arachidonic acid metabolism and human neutrophil migration in asthma. *Prostaglandins Leukotrienes and Essential Fatty Acids* 1990; **41:** 131–138.
12. Taylor MB, Zweiman B, Moskovitz AR, von Allen C, Atkins PC. Platelet-activating factor- and leukotriene B4-induced release of lactoferrin from blood neutrophils of atopic and non atopic individuals. *J Allergy Clin Immunol* 1990; **86:** 740–748.
13. Carlson M, Hakansson L, Petersen C, Stalenheim G, Venge P. Secretion of granule proteins from eosinophils and neutrophils is increased in asthma. *J Allergy Clin Immunol* 1991; **87:** 27–33.
14. Pacheco Y, Hosni R, Chabannes B, *et al.* Leukotriene B4 level in stimulated blood neutrophils and alveolar macrophages from healthy and asthmatic subjects. Effect of beta-2 agonist therapy. *Eur J Clin Investigation* 1992; **22:** 732–739.
15. Kallenbach J, Baynes R, Fine B, Dajee D, Bezwoda W. Persistent neutrophil activation in mild asthma. *J Allergy Clin Immunol* 1992; **90:** 272–274.
16. Meltzer S, Goldberg B, Lad P, Easton J. Superoxide generation and its modulation by adenosine in the neutrophils of subjects with asthma. *J Allergy Clin Immunol* 1989; **83:** 960–966.
17. Adelroth E, Rosenhall L, Johansson SA, Linden M, Venge P. Inflammatory cells and eosinophilic activity in asthmatics investigated by bronchoalveolar lavage: The effects of antiasthmatic treatment with budesonide or terbutaline. *Am Rev Respir Dis* 1990; **142:** 91–99.
18. Clark RAF, Gallin JI, Fauci AS. Effects of *in vivo* prednisone on *in vitro* eosinophil and neutrophil adherence and chemotaxis. *Blood* 1979; **53:** 633–641.
19. Gin W, Shaw RJ, Kay AB. Airways reversibility after prednisolone therapy in chronic asthma is associated with alterations in leukocyte function. *Am Rev Respir Dis* 1985; **132:** 1199–1203.
20. Gin W, Kay AB. The effect of corticosteroids on monocyte and neutrophil activation in bronchial asthma. *J Allergy Clin Immunol* 1985; **76:** 675–682.
21. Shiratsuki N, Uyama O, Kitada O, *et al.* Effects of hydrocortisone and aminophylline on plasma leukotriene C4 levels in patients during an asthmatic attack. *Prostaglandins Leukotrienes and Essential Fatty Acids* 1990; **40:** 285–289.
22. Freeland HS, Pipkorn U, Schleimer RP, *et al.* Leukotriene B4 as a mediator of early and late reactions to antigen in humans: The effect of systemic glucocorticoid treatment *in vivo*. *J Allergy Clin Immunol* 1989; **83:** 634–642.
23. American Thoracic Society. Lung function testing: Selection of reference values and interpretative strategies. *Am Rev Respir Dis* 1991; **144:** 1202–1218.
24. Armitage P. *Statistica medica.* 5th ed. Milano: Feltrinelli; 1981.
25. Schleimer RP, Freeland HS, Peters SP, Brown KE, Dorse CP. An assessment of the effects of glucocorticoids on degranulation, chemotaxis, binding to vascular endothelial and formation of leukotriene B4 by purified human neutrophils. *J Pharmacol Exp Ther* 1989; **250:** 598–605.
26. Paggiaro PL, Dente FL, Azzara' A, *et al.* Abnormal migratory activity of peripheral neutrophils from asthmatic patients and its modulation by inhaled glucocorticoids. *J Invest Allergol Clin Immunol* 1993; **3:** 237–244.
27. Carmichael J, Paterson IC, Diaz P, Crompton GK, Kay AB, Grant IWB. Corticosteroid resistance in chronic asthma. *Br Med J* 1981; **282:** 1419–1422.
28. Pin I, Gibson GG, Kolendowicz R, *et al.* Use of induced sputum cell counts to investigate airway inflammation in asthma. *Thorax* 1992; **47:** 25–29.
29. Lacoste JY, Bousquet J, Chanez P, *et al.* Eosinophilic and neutrophilic inflammation in asthma, chronic bronchitis, and chronic pulmonary disease. *J. Allergy Clin Immunol* 1993; **92:** 537–548.

**Received 17 February 1995; accepted in revised form 4 April 1995**
